# Mysm1 epigenetically regulates the immunomodulatory function of adipose‐derived stem cells in part by targeting miR‐150

**DOI:** 10.1111/jcmm.14281

**Published:** 2019-03-20

**Authors:** Yu‐Han Wang, Xiao‐Hui Huang, Yan‐Mei Yang, Youdi He, Xiao‐Hui Dong, Hui‐Xin Yang, Lei Zhang, Yan Wang, Jin Zhou, Changyong Wang, Xiao‐Xia Jiang

**Affiliations:** ^1^ Department of Neural Engineering and Biological Interdisciplinary Studies Institute of Military Cognition and Brain Sciences, Academy of Military Medical Sciences Beijing P.R. China; ^2^ Anhui Medical University Hefei Anhui China; ^3^ Department of Stomatology Chinese PLA General Hospital Beijing P.R. China; ^4^ Department of Neurology Beijing Chaoyang Hospital, Capital Medical University Beijing China; ^5^ College of Agroforestry Engineering and Planning, Tongren University Tongren, Guizhou China

**Keywords:** adipose‐derived stem cells, miR‐150, Mysm1, nitric oxide

## Abstract

Adipose‐derived stem cells (ASCs) are highly attractive for cell‐based therapies in tissue repair and regeneration because they have multilineage differentiation capacity and are immunosuppressive. However, the detailed epigenetic mechanisms of their immunoregulatory capacity are not fully defined. In this study, we found that Mysm1 was induced in ASCs treated with inflammatory cytokines. Adipose‐derived stem cells with Mysm1 knockdown exhibited attenuated immunosuppressive capacity, evidenced by less inhibition of T cell proliferation, more pro‐inflammatory factor secretion and less nitric oxide (NO) production in vitro. Mysm1‐deficient ASCs exacerbated inflammatory bowel diseases but inhibited tumour growth in vivo. Mysm1‐deficient ASCs also showed depressed miR‐150 expression. When transduced with Mysm1 overexpression lentivirus, ASCs exhibited enhanced miR‐150 expression. Furthermore, Mysm1‐deficient cells transduced with lentivirus containing miR‐150 mimics produced less pro‐inflammatory factors and more NO. Our study reveals a new role of Mysm1 in regulating the immunomodulatory activities of ASCs by targeting miR‐150. These novel insights into the mechanisms through which ASCs regulate immune reactions may lead to better clinical utility of these cells.

## 1 INTRODUCTION

Adipose‐derived stem cells (ASCs) are similar to bone marrow‐derived stem cells (BMSCs) in their ability to differentiate into multiple cell types, including bone, cartilage, adipocytes and neurons.^1^, ^2^ Additionally, ASCs are immunosuppressive and express similar surface markers to BMSCs.^6^ Unlike BMSCs, clinically applicable numbers of ASCs can easily be obtained from adipose tissue collected through minimally invasive procedures such as lipoplasty.^7^ Due to these properties, ASCs are appealing for use in cell‐based therapies for tissue repair and regeneration.

Allogeneic ASCs are immune privileged and have immunomodulatory capabilities. In vitro, ASCs inhibit the proliferation and function of activated immune cells through cell‐cell binding and paracrine signalling.^8^ In vivo, ASCs have demonstrated therapeutic potential in numerous immune‐mediated conditions in both pre‐clinical and clinical studies, including graft‐vs‐host disease (GvHD) and chronic inflammatory autoimmune diseases.^9^, ^10^ There are several possible mechanisms through which ASCs function to suppress immunity. A series of factors and molecules produced by ASCs, such as prostaglandin (PG) E2,^13^ transforming growth factor‐β (TGF‐β)^14^ and interleukin (IL)‐10,^14^, ^15^ have been shown to be critical for their immunoregulatory functions. While many of these factors have been well characterized, much remains unknown about the immunomodulatory function and therapeutic efficacy of ASCs.

Mysm1, a histone deubiquitinase, mediates the deubiquitination of lysine 119 (K119) of histone H2A,^16^ and removes K63 polyubiquitins attached to TRAF3 and TRAF6.^17^ We and several other groups have previously demonstrated that Mysm1 plays a crucial role in stem cell maintenance and immune cell development and function.^18^, ^19^ Mysm1 can control essential lineage‐specific developmental regulators and miRNA expression at a transcriptional level, and it regulates the p53 stress response pathway in a cell‐specific manner.^25^, ^26^ Despite these observations, knowledge of the biological functions of Mysm1 remains incomplete and its role in ASC immunoregulatory function has not been investigated.

In the present study, we demonstrate that Mysm1‐deficient ASCs showed attenuated inhibition of T cell proliferation in vitro, while exacerbating inflammatory bowel diseases and inhibiting tumour growth in vivo. Further mechanistic studies revealed that Mysm1 regulates the immunomodulatory capacity of ASCs by targeting miR‐150 expression. Taken together, our data reveal a novel role of Mysm1 in regulating the immunomodulatory activities of ASCs.

## 2 MATERIALS AND METHODS

### 2.1 Animals

Groups of 3‐4‐week‐old and 8‐12‐week‐old C57BL/6 mice were obtained from the Laboratory Animal Center of the Academy of Military Medical Sciences of China (Beijing). Mysm1‐deficient (KO) mice were generated as described previously.^18^ In all experiments, age‐ and sex‐matched wild type (WT) littermates were used for controls. Mice were maintained in a pathogen‐free barrier facility. All animal experiments were performed according to the ‘Guide for the Care and Use of Laboratory Animals’ approved by the Beijing Institute of Military Cognition and Brain Sciences. The institutional Ethics Review Committee for Animal Experimentation approved all experimental protocols.

### 2.2 ASC isolation and culture

Wild type and Mysm1^‐^deficient mice (3‐4 weeks old) were euthanized by cervical dislocation and sterilized in 75% ethanol for 5 minutes. Adipose‐derived stem cells were obtained from inguinal subcutaneous adipose tissue with abdomens facing up. The collected tissue was rinsed with phosphate buffered solution (PBS) and minced, followed by digestion with 1 mg/mL collagenase type IV (Sigma‐Aldrich, St. Louis, MO) and 1 mg/mL dispase (Sigma‐Aldrich) for 35 minutes at 37°C with agitation. Cells were added to α‐MEM (Gibco, Carlsbad, CA) containing 10% fetal bovine serum (FBS) (Gibco) to stop digestion and filtered through a 40 μm cell strainer (Biologix, Lenexa, KS) to generate single‐cell suspensions. After centrifugation at 400 g for 5 minutes, cells were resuspended in α‐minimum essential medium (α‐MEM) containing 10% FBS and cultured at 37°C in 5% CO_2_. Upon 80%‐90% confluence, cells were subcultured in a ratio of 1:3.

### 2.3 Immunofluorescence staining

For immunofluorescence, cells were fixed in 4% formaldehyde for 20 minutes at room temperature, permeabilized with 0.3% Triton X‐100 in PBS for 10 minutes and then blocked with 2% bovine serum albumin and 0.05% sodium azide in PBS for 1 hour at room temperature to block non‐specific antibody binding. Subsequently, cells were incubated with the primary antibodies overnight at 4°C. Cells were then washed three times for 10 minutes with PBS and incubated for 1 hour at room temperature with Alexa Fluor 488‐conjugated secondary antibody (Invitrogen, Carlsbad, CA). Then the cells were counterstained with 4′,6‐diamidino‐2‐phenylindole in D‐PBS (Sigma‐Aldrich) and analyzed under a Zeiss confocal microscope.

### 2.4 Carboxy fluorescein diacetate succinimidyl ester labelling

Splenocytes were isolated from C57BL/6 mice (8‐12 weeks old). CD3^+ ^T cells were selected using a CD3e MicroBead Kit (Miltenyi Biotec, Bergisch Gladbach, Germany), and then labelled with 5 μmol/L carboxy fluorescein diacetate succinimidyl ester (CFSE; Invitrogen, Carlsbad, CA) for 7 minutes at 4°C. Labelling was terminated according to the manufacturer's protocol. After washing, cells were activated with 50 ng/mL phorbol myristate acetate (PMA) and 1 μg/mL ionomycin (Sigma‐Aldrich) for 16 hours, and then co‐cultured with or without ASCs for 48 hours. Cell division, as indicated by reduction of fluorescence intensity, was analysed by flow cytometry.

### 2.5 Quantitative RT‐PCR

Total RNA was extracted with TRIzol (Sigma‐Aldrich) and reverse transcribed into cDNA with a reverse transcriptase kit (Toyobo, Osaka, Japan). cDNA was used as a template in quantitative PCR with Synergy Brands Synergy Brands (SYBR) Green (Toyobo) to determine specific gene expression. Total mRNA was normalized to endogenous glyceraldehyde‐3‐phosphate dehydrogenase (GAPDH) mRNA. Primer pairs were as follows: Mysm1: GATGCAGAAGCAGCATACCA (forward) and CCTCCACAGACAAATGCTCA (reverse); inducible nitric oxide synthases (iNOS): CAGCTGGGCTGTACAAACCTT (forward) and CATTGGAAGTGAAGCGTTTCG (reverse); IL‐10: CCAAGCCTTATCGGAAATGA (forward) and TCTCACCCAGGGAATTCAAA (reverse); interferon‐gamma (IFNγ): GGTCAACAACCCACAGGTC, (forward) and GACTCCTTTTCCGCTTCCT (reverse); IL‐1β: CATTAGACAACTGCACTACAGG (forward) and GTTCTCCTTGTACAAAGCTCAT (reverse); IL‐6: AGATAAGCTGGAGTCACAGAAGGAG (forward) and CGCACTAGGTTTGCCGAGTAG (reverse); transforming growth factor‐β1 (TGF‐β1): TTGACGTCACTGGAGTTGTA (forward) and CCACGTGGAGTTTGTTATCT (reverse); and GAPDH: ACAATGAATACGGCTACAG (forward) and GTCCAGGGTTTCTTACTC (reverse).

### 2.6 Lentivirus production and transduction

Recombinant lentiviral vectors containing Mysm1 or miR‐150 were purchased from Genechem (Shanghai, China). Adipose‐derived stem cells or C3H/10T1/2 cells were transduced as described in our previous publications.^18^, ^25^

### 2.7 Chromatin immunoprecipitation

Chromatin was immunoprecipitated according to the manufacturer's instructions (Cell Signaling Technology, Danvers, MA). Briefly, cell suspensions were crosslinked with 1% (vol/vol) formaldehyde. Chromatin was isolated, digested by mung bean nuclease (MNase), sheared by sonication and immunoprecipitated with antibodies. Immunoprecipitated DNA was washed and eluted according to the manufacturer's instructions. Eluted DNA and sheared input material was analyzed by quantitative PCR. Primer pairs for miR‐150 promoter regions were as follows: miR‐150 prom 9: AGGTTATCAC TATGCAGACA (forward) and CAGGGTTTCT CTGTGTAACA (reverse); miR‐150 prom 10: TCTTGCAAAA CAAACAACCA (forward) and TGGAGGGCTT TTCTAACAAG (reverse).

### 2.8 Western blot

Cells were lysed with lysis buffer and protein samples were separated on 12% SDS‐polyacrylamide gel, and then the proteins were transferred to 0.45 μm polyvinylidene fluoride blotting membranes. The membrane was blocked in 5% non‐fat dry milk for 1 hour, were then probed with primary antibodies against the proteins of interest in blocking solution overnight at 4°C, washed and then incubated in horseradish peroxidase (HRP)‐conjugated secondary antibodies for 1 hour at room temperature. Finally, enhanced chemiluminescence substrate (Thermo Fisher, Waltham, MA) was added to the membranes and the proteins were assayed according to manufacturer instructions. Antibodies against GAPDH, Mysm1 were purchased from Cell Signaling Technology, Inc.

### 2.9 Induction of acute colitis

Acute colitis was induced in C57BL/6 mice by administering 3% dextran sodium sulphate (DSS; molecular weight 40 000 Da; Sigma‐Aldrich) from day 0 to day 7 in drinking water. On day 1, WT and Mysm1 KO ASCs were injected intraperitoneally in DSS‐treated animals. Colitis severity was assessed daily by scoring (0‐4) the clinical disease activity through evaluation of stool consistency, presence of faecal blood and weight loss. Mice with acute colitis were euthanized on day 8. The entire colon was removed from the caecum to the anus, and colon length and weight were measured as indirect inflammation markers. The macroscopic colonic damage score was assessed based on the grade of tissue adhesion, presence of ulceration and wall thickness.

### 2.10 Mouse melanoma model

B16‐F0 murine melanoma cells (CRL‐6322; ATCC, Manassas, VA) were expanded in complete DMEM in vitro. Each mouse was injected with 5 × 10^5^ B16‐F0 cells in 100 μL PBS intramuscularly on the left thigh, with or without co‐injection of WT or Mysm1‐deficient ASCs (1 × 10^6 ^per mouse). Mice were observed daily and euthanized when tumours began to significantly affect mobility. Melanoma tumours were then excised and weighed. Each experimental group included at least five mice.

### 2.11 Statistical Analysis

All data were analyzed with Prism 5.0 software (GraphPad Software, San Diego, CA) and are presented as the means ± SDs. Statistical significance was assessed by unpaired two‐tailed Student's *t* tests (*P < 0.05; **P < 0.01).

## 3 RESULTS

### 3.1 Inflammatory cytokines induce Mysm1 expression in ASCs

**Figure 1:**
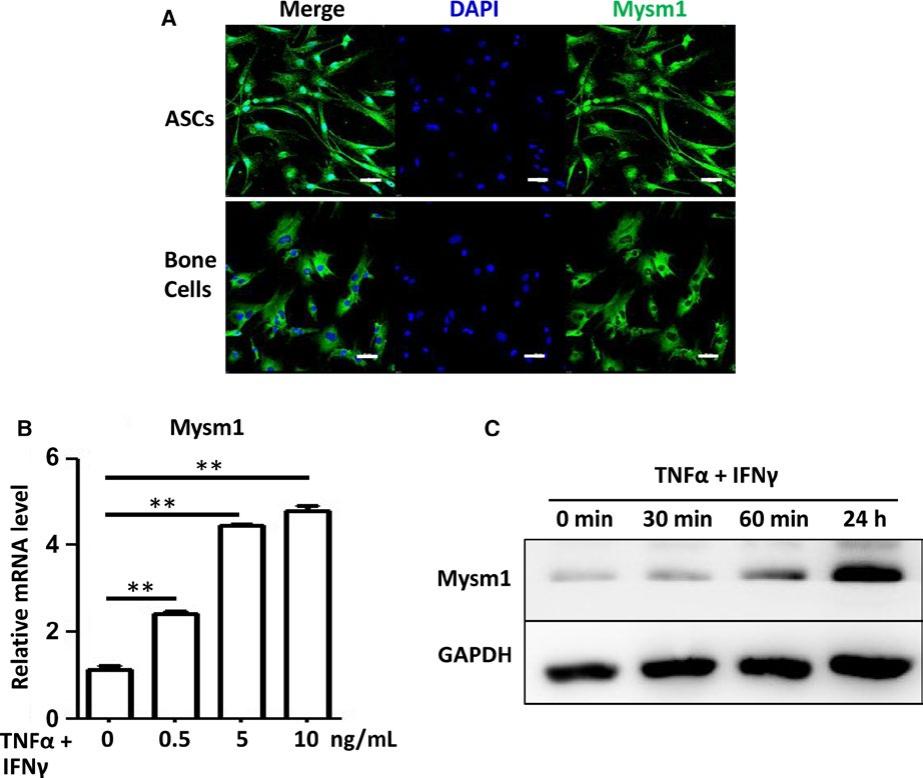
Inflammatory cytokines induce Mysm1 expression. A, Immunofluorescence analysis of Mysm1 expression in murine‐derived adipose‐derived stem cells (ASCs) and bone cells. Scale bars: 50 µm. B, Adipose‐derived stem cells were treated with tumour necrosis factor‐α (TNF‐α) plus IFNγ for 12 h at different concentrations and then collected in TRIzol. Mysm1 mRNA levels were determined with quantitative RT‐PCR. C, Adipose‐derived stem cells were treated with 10 ng/mL TNF‐α and 10 ng/mL IFNγ for 30 min, 60 min and 24 h, then Mysm1 protein levels were determined by Western blot. **P < 0.01

Mysm1 plays essential roles in stem cell maintenance and immune cell function. Mysm1 activity has been reported in the nucleus^16^ and cytoplasm^17^ with cell‐specific properties. Adipose‐derived stem cells are stem cells with immunomodulatory capacities. To investigate the effect of Mysm1 on ASCs, the expression levels of Mysm1 in ASCs was examined. Immunofluoresence staining (Figure 1A) showed that under basal conditions, Mysm1 was localized in both nucleus and cytoplasm of ASCs, whereas adherent cells isolated from murine bone expressed Mysm1 in the cytoplasm. To further determine the effect of Mysm1 on the immunomodulatory function of ASCs, Mysm1 expression in ASCs treated with inflammatory cytokines was analyzed. As shown in Figure 1B, Mysm1 mRNA levels increased in a dose‐dependent manner with tumour necrosis factor‐α (TNF‐α) and IFNγ stimulation. Similarly, protein expression was also induced with TNF‐α and IFNγ treatment (Figure 1C). These data indicate that Mysm1 may be involved in the immunomodulatory activity of ASCs.

### 3.2 Mysm1 knockdown attenuates the immunosuppressive capacity of ASCs

**Figure 2:**
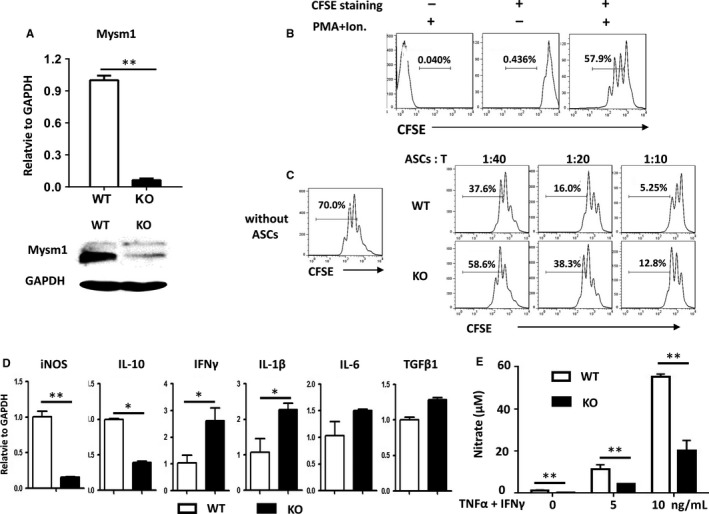
Properties of adipose‐derived stem cells (ASCs) with Mysm1 knockdown. A, Adipose‐derived stem cells were isolated from wild type (WT) and Mysm1‐deficient (KO) mice. Mysm1 mRNA levels (top) were determined by quantitative RT‐PCR, and protein expression (bottom) was examined by Western blot. B, Flow cytometry analysis of CD3^+^ T cell proliferation as indicated by reduced carboxy fluorescein diacetate succinimidyl ester (CFSE) intensity. CD3^+^ T cells were isolated from murine spleens with CD3e MicroBead Kits and labelled with or without CFSE. CD3^+^ T cells were stimulated with or without PMA (50 ng/mL) plus ionomycin (1 µg/mL) for 72 h, and then cells were collected for flow cytometry analysis. C, Attenuated inhibition of T cell proliferation by KO ASCs. CD3^+^ T cells were labelled with CFSE and stimulated with PMA (50 ng/mL) plus ionomycin (1 µg/mL) for 24 h, and then cultured alone (left), with WT ASCs, or with KO ASCs at different ratios (ASCs:T cells) (right). After 48 h, cells were analyzed by flow cytometry for T cell proliferation as indicated by reduced CFSE intensity. Data are representative of two independent experiments. D, mRNA levels of inflammatory cytokines in WT and KO ASCs were determined by quantitative RT‐PCR. E, After treating WT and KO ASCs with tumour necrosis factor‐α (TNF‐α) plus IFNγ for 24 h, the amount of nitrate in the supernatant was determined by a Griess test. *P < 0.05 and **P < 0.01

To test the involvement of Mysm1 in ASC immunomodulatory activity, ASCs from Mysm1 KO and WT mice were isolated. The deficient expression of Mysm1 in KO ASCs was confirmed by quantitative RT‐PCR and Western blot analysis (Figure 2A). Surface markers and cell cycle were examined by flow cytometry and no significant differences were observed between cultures of KO ASCs and their WT counterparts (data not shown). Next, T cell proliferation was used as an immune response model, in which a reduction of CFSE intensity was measured to determine T cell proliferation (Figure 2B). As shown in Figure 2C, both WT and KO ASCs directly inhibited T cell proliferation in a dose‐dependent manner, but KO ASCs were less efficient, evidenced by a lesser reduction in CFSE intensity. To better characterize the function of Mysm1, inflammatory cytokine expression in WT and KO ASCs was analyzed. Quantitative RT‐PCR data (Figure 2D) revealed that under basal culture condition and compared to WT counterparts, KO ASCs had higher expression of the inflammatory genes IFN_γ_ and IL‐1β, and lower expression of the anti‐inflammatory gene IL‐10 and much less iNOS. Additionally, no matter without or with TNF‐α and IFNγ stimulation, KO ASCs exhibited significantly lower nitric oxide (NO) production (Figure 2E).

### 3.3 KO ASCs exacerbated dextran sulfate sodium‐induced colitis but suppressed tumour growth in vivo

**Figure 3:**
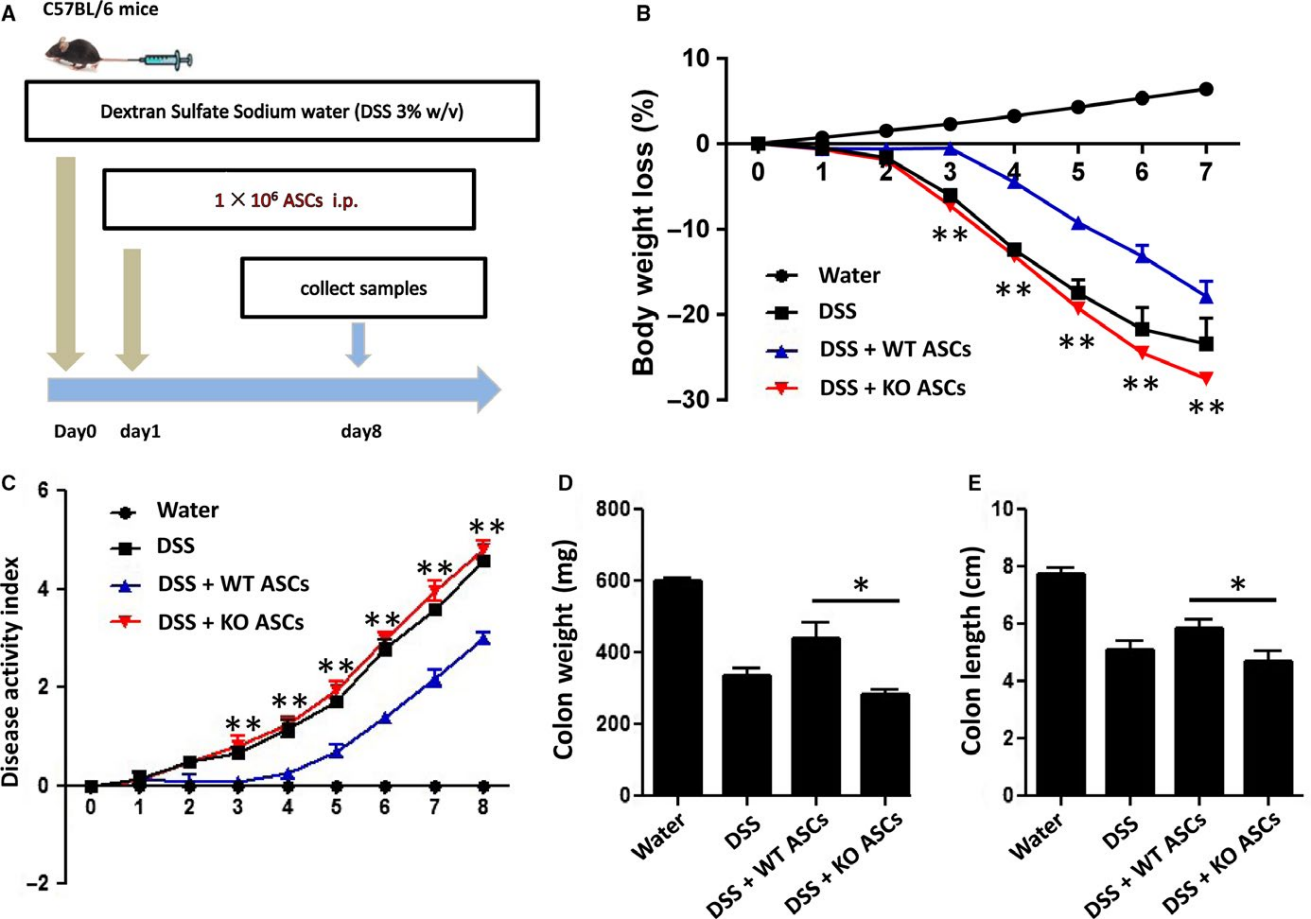
Mysm1‐deficient adipose‐derived stem cells (ASCs) exacerbated dextran sodium sulphate (DSS)‐induced colitis. A, Schematic representation of mouse colitis experiments. Mice received 3% DSS in drinking water from day 0 to day 8. Adipose‐derived stem cells (1 × 10^6^/mouse) were infused intraperitoneally on day 1. Weight loss (B) and disease activity scores (C) were observed daily. Colon weight (D) and colon length (E) were measured on day 8. Control mice received no DSS in drinking water. n = 6 mice/group. *DSS + WT ASCs vs DSS + KO ASCs, *P < 0.05 and **P < 0.01

Next, the physiological function of KO ASCs was investigated in an experimental model of acute colitis induced by oral dextran sulphate sodium (DSS) administration. After 8 days, mice receiving an oral administration of 3% DSS exhibited a significant increase in the disease activity index, characterized by acute colitis, bloody diarrhoea and sustained weight loss (Figure 3A‐E). Subsequent treatment with WT ASCs increased survival rate, ameliorated weight loss and improved the disease severity. Conversely, treatment with KO ASCs exacerbated DSS‐induced colitis, leading to increased disease severity characterized by further weight loss, diarrhoea and bloody stools and signs of colon damage (Figure 3B‐E).

**Figure 4:**
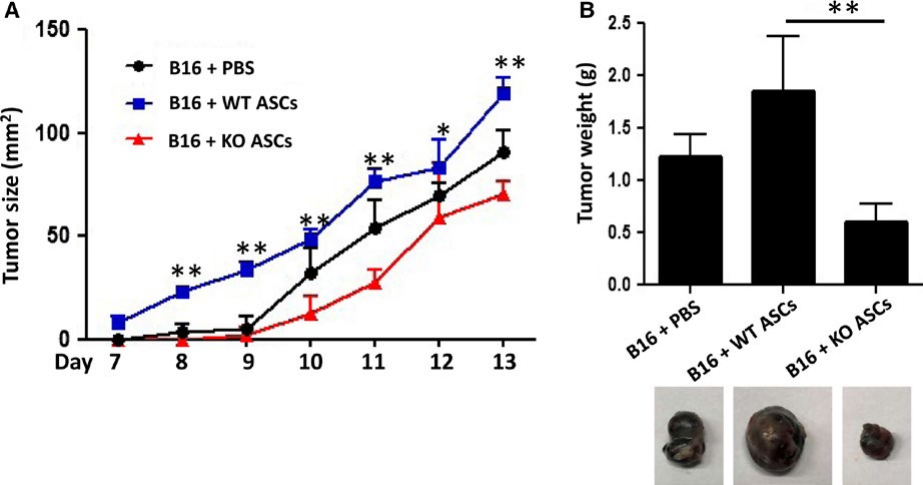
Mysm1‐deficient adipose‐derived stem cells (ASCs) suppress tumour growth in vivo. On day 1, 5 × 10^5^ B16‐F0 cells were injected subcutaneously to the back of C57BL/6 mice, with or without co‐injection of WT or KO ASCs (1 × 10^6^ cells per mouse). Seven days later, tumour size was measured daily (A). On day 13, mice were euthanized and the tumours were weighed (B, top), and pictures of the representative tumour of each group were taken under a microscope (B, bottom). Each treatment group included five mice, and data are representative of three independent experiments. Wild type (WT) ASCs vs KO ASCs, *P < 0.05 and **P < 0.01

Previous studies have shown that mesenchymal stem cells (MSCs) with attenuated immunosuppressive capacities can inhibit tumour growth.^27^, ^28^ Compared to WT counterparts, KO ASCs are less immunosuppressive both in vitro and in vivo. Thus a murine melanoma model was used to determine the effect of KO ASCs on tumour growth in vivo. B16‐F0 melanoma cells were co‐administered with WT ASCs or KO ASCs and the resultant tumours were weighed after 13 days. In contrast to WT ASCs that promoted tumour growth, infusion of KO ASCs was found to significantly inhibit tumour growth (Figure 4A,B). Therefore, modulation of ASCs could provide a novel strategy for tumour immunotherapy.

### 3.4 Mysm1 epigenetically regulates miR‐150 expression in ASCs

**Figure 5:**
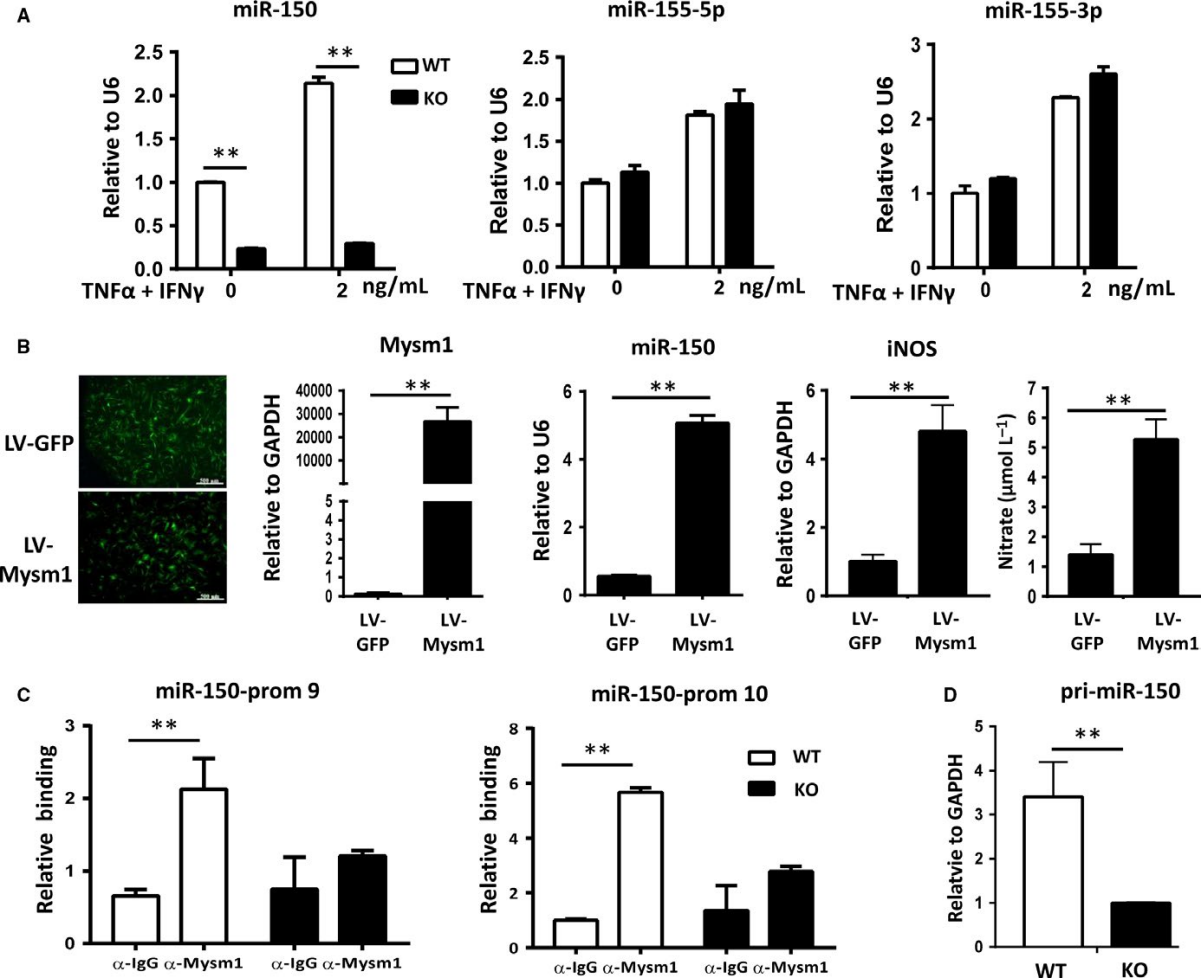
Mysm1 epigenetically regulates miR‐150 expression. A, Wild type (WT) and KO adipose‐derived stem cells (ASCs) were treated with tumour necrosis factor‐α (TNF‐α) plus IFNγ for 24 h and cells were collected in TRIzol. Levels of miR‐150, miR‐155‐5p, and miR‐155‐3p were determined with quantitative RT‐PCR. B, Ectopic expression of Mysm1 in KO ASCs. Cells were transduced with lentivirus containing GFP (LV‐GFP) or Mysm1 (LV‐Mysm1) (left). Quantitative RT‐PCR results show increased expression of Mysm1, miR‐150 and inducible nitric oxide synthases and Griess test show the increase amount of nitrate in KO ASCs transduced with LV‐Mysm1. Data are representative of three independent experiments and shown as the mean ± SD. C, Chromatin immunoprecipitation assays of WT ASCs and KO ASCs via an Mysm1 antibody probing for the pri‐miR‐150 promoter sequence or an IgG nonspecific antibody. D, Quantitative RT‐PCR result shows decreased expression of pri‐miR‐150 in KO ASCs. *P < 0.05 and **P < 0.01

miR‐150 is an important regulator of differentiation and activation of immune cells. Our previous studies have demonstrated that Mysm1 regulates miR‐150 expression and is involved in B1a cell proliferation.^25^ Here, the expression of miR‐150 in KO ASCs or an Mysm1 knockdown murine MSC line was significantly lower than that in their WT counterparts (Figure 5A). The expression of miR‐155‐5p and miR‐155‐3p was comparable in WT and KO ASCs, although miR‐155 has been reported to regulate immune modulatory properties of MSCs.^29^ We therefore focused on miR‐150 for further studies. To investigate how Mysm1 might regulate miR‐150 in ASCs, we first transduced KO ASCs with Mysm1‐expressing lentivirus (LV‐Mysm1). As shown in Figure 5B, most cells were green fluorescent protein (GFP) positive, indicating high transduction efficiency. Quantitative RT‐PCR data confirmed the over expression of Mysm1. miR‐150 level was also examined in KO ASCs with Mysm1 overexpression. miR‐150 expression was significantly increased in KO ASCs with Mysm1 overexpression compared to that of control transduced counterparts (Figure 5B). Next, we set out to examine whether Mysm1 regulates the transcription of miR‐150 and investigated the association of Mysm1 with the miR‐150 promoter locus by using chromatin immunoprecipitation (ChIP) assays. PCR primer pairs encompassing the pri‐miR‐150 promoter region were used. Immunoprecipitation with the Mysm1‐specific antibody enriched the sequences located at pri‐miR‐150 promoter in WT ASCs, but not in KO ASCs (Figure 5C), which might account for the lower expression of pri‐miR‐150 in KO ASCs (Figure 5D).

### 3.5 miR‐150 regulates iNOS expression

**Figure 6:**
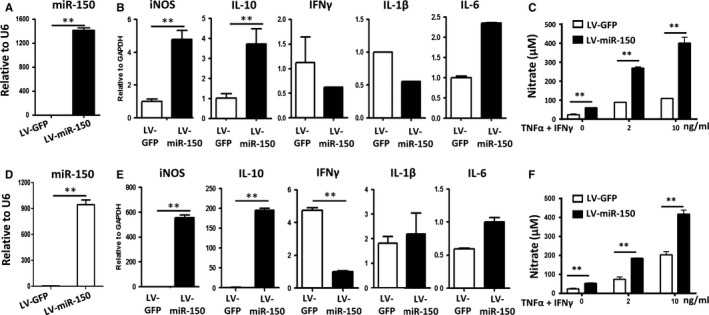
miR‐150 regulates inducible nitric oxide synthases (iNOS) expression. A, KO adipose‐derived stem cells (ASCs) were transduced with lentivirus containing GFP (LV‐GFP) or LV‐miR‐150, and miR‐150 expression was detected by quantitative RT‐PCR. B, mRNA levels of inflammatory cytokines in KO ASCs transduced with LV‐GFP or LV‐miR‐150 was determined by quantitative RT‐PCR. C, KO ASCs transduced with LV‐GFP or LV‐miR‐150 were treated with tumour necrosis factor‐α (TNF‐α) plus IFNγ at different concentrations for 24 h, then levels of nitrate in the supernatant was measured by a Griess test. D, After C3H/10T1/2 cells were transduced with LV‐GFP or LV‐miR‐150, miR‐150 expression was detected by quantitative RT‐PCR. E, mRNA levels of inflammatory cytokines in C3H/10T1/2 cells transduced with LV‐GFP or LV‐miR‐150 was determined by quantitative RT‐PCR. F, C3H/10T1/2 cells transduced with LV‐GFP or LV‐miR‐150 were treated with TNF‐α plus IFNγ at different concentrations for 24 h, then levels of nitrate in the supernatant was measured by a Griess test. **P < 0.01

**Figure 7:**
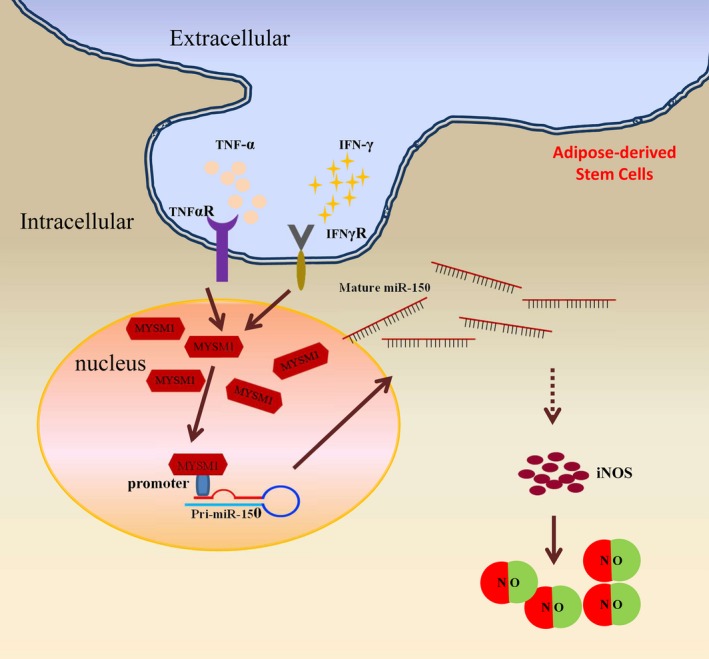
A proposed model of the mechanism by which Mysm1 regulates the immunomodulatory effect of adipose‐derived stem cells (ASCs). Mysm1 is induced by tumour necrosis factor‐α (TNF‐α) and IFNγ in ASCs. Mysm1 then promotes miR‐150 transcription, which enhances inducible nitric oxide synthases (iNOS) production. Nitric oxide (NO) is catalyzed by iNOS that is essential for the immunosuppressive capacity of ASCs

To further investigate the role of Mysm1 in controlling miR‐150 expression as part of the immunomodulatory function of ASCs, we performed a rescue assay in KO ASCs with a lentivirus vector expressing miR‐150. GFP expression indicated high transduction efficiency (data not shown) and quantitative RT‐PCR data confirmed the overexpression of miR‐150 in KO ASCs (Figure 6A). Higher expression of iNOS was found in KO ASCs with miR‐150 overexpression (Figure 6B). And when stimulated with TNF‐α and IFNγ, KO ASCs transduced with miR‐150 mimics produced much more NO (Figure 6C). Inducible nitric oxide synthases is essential for MSC‐induced immunosuppression. Similarly, the murine MSC cell line C3H/10T1/2 transduced with miR‐150 mimics (Figure 6D) also showed higher level of iNOS (Figure 6E), and exhibited more NO production (Figure 6F) with TNF‐α and IFNγ treatment. However, miR‐150 transduction did not affect the expression of surface markers programmed cell death 1 ligand 1(PD‐L1), vascular cell adhesion molecule 1(VCAM‐1) or intercellular adhesion molecule‐1 (ICAM‐1) (data not shown). These data indicate that Mysm1 epigenetically regulates miR‐150, which leads to enhanced iNOS expression and thus more NO production (Figure 7).

## 4 DISCUSSION

In addition to their use in tissue repair and regenerative medicine, ASCs have been utilized in treating immune disorders due to their immunomodulatory properties.^9^, ^10^ A series of factors are known to be critical for ASC immunoregulation. The present study provides the first report on alteration of ASC immunomodulatory function by the epigenetic molecule Mysm1, which regulates miR‐150.

Previous studies have shown that Mysm1 controls the development of several hematopoietic lineages, including B,^18^ T,^24^ natural killer,^30^ and dendritic cells.^31^ This is attributed to the function of Mysm1 in the direct regulation of lineage‐specific transcription factors. In addition to localizing in the nucleus and being a key component of epigenetic signalling machinery, Mysm1 can interact with and inactivate TRAF3 and TRAF6, and thus inhibit PRR pathways in the cytoplasm.^17^ Similar to MSCs, the immunosuppressive function of ASCs is elicited by proinflammatory cytokines. In the present study, we show that Mysm1 is induced by inflammatory cytokines in ASCs similar to in macrophages. However, while localized to the cytoplasm of macrophages,^17^ Mysm1 is located in both the nuclei and cytoplasm of ASCs, and epigenetically regulates the expression of miR‐150. Despite these novel findings, more studies are required to understand the activity of Mysm1 in the cytoplasm of ASCs.

Mesenchymal stem cell‐based immune regulation mainly occurs through paracrine effects by the production of soluble factors, including NO,^32^ prostaglandin E2 (PGE2)^33^, ^34^ indoleamine 2,3‐dioxygenase (IDO),^35^ and TGF‐β,^36^ but may also occur through direct cell‐cell contact.^37^, ^38^ Our previous studies revealed that suppressor of cytokine signalling 1 (SOCS1)^39^ and the ubiquitin‐modifying enzyme A20^28^ are also involved in the immunoregulatory function of MSCs. Recent studies have also shown that miRNAs critically regulate immune responses. Xu et  al^29^ reported that miR‐155 was induced by inflammatory cytokines and inhibited the immunosuppressive capacity of MSCs by reducing iNOS expression. In the present study, miR‐155 was also induced in ASCs treated with inflammatory cytokines; however, the expression level of miR‐155 was unchanged for WT and KO ASCs. In contrast to miR‐155, the expression of miR‐150 in KO ASCs was significantly lower than that in WT ASCs. In addition, the expression of miR‐150 is also decreased in human placental derived MSCs with Mysm1 knockdown and in murine MSC C3H/10T1/2 cells with Mysm1 knockdown (data not shown). Furthermore, we showed that Mysm1 targets the promoter region of pri‐miR‐150, and over‐expression of Mysm1 in ASCs leads to significant upregulation of miR‐150. NO production is catalyzed by NO synthases and NO is essential for the immunosuppressive capacity of ASCs.^32^, ^40^ Compared with those in WT counterparts, the supernatant nitrate concentration and iNOS mRNA expression were significantly lower in KO ASCs. When KO ASCs or murine MSC C3H/10T1/2 cells were transduced with miR‐150 mimics, NO production was dramatically increased.

## 5 CONCLUSIONS

This study reveals that Mysm1 regulates the immunosuppressive capacity of ASCs by targeting miR‐150, and thus uncovers a previously undescribed role of Mysm1 in regulating the immunomodulatory activities of ASCs. These novel insights into the mechanisms through which ASCs regulate immune reactions may help to improve the clinical utility of these cells in many inflammation related diseases.

## ETHICS APPROVAL AND CONSENT TO PARTICIPATE

All experimental animal protocols for this study are in accordance with the national guidelines for the use of animals in scientific research. Additional approval was granted by the Animal Care and Use Committee of the Academy of Military Medical Sciences.

## CONFLICT OF INTEREST

The authors declare no conflicts of interest.

## AUTHORS' CONTRIBUTIONS

XXJ and CW conceived and designed the experiments. YHW, XHH, YMY, YH, XHD, HXY, LZ and YW performed the experiments. YHW, XHH, YMY, YH, XHD, HXY, LZ, YW, JZ, XXJ and CW analyzed experimental data. XXJ and CW wrote the manuscript. All authors read and approved the manuscript.
